# Quantitative Evidence of Wear-Off Effect at the End of the Intravenous IgG (IVIG) Dosing Cycle in Primary Immunodeficiency

**DOI:** 10.1007/s10875-016-0243-z

**Published:** 2016-02-24

**Authors:** Mikhail A. Rojavin, Alphonse Hubsch, John-Philip Lawo

**Affiliations:** Clinical Research and Development, CSL Behring LLC, King of Prussia, PA USA; Medical Affairs, CSL Behring AG, Berne, Switzerland; Clinical Research and Development, CSL Behring GmbH, Marburg, Germany

**Keywords:** Immunoglobulin replacement therapy, ivig, primary immunodeficiency, scig, wear-off, end-dose effect, well-being

## Abstract

**Purpose:**

Intravenous IgG (IVIG) treatment wear-off is commonly experienced by patients, who report increased susceptibility to infection, and decreased quality of life towards the end of their 3- or 4-week dosing cycle, when serum IgG levels approach their trough. We quantified IVIG wear-off in terms of treatment efficacy and patient well-being.

**Methods:**

Data were collected from patients enrolled in three Phase III trials of Sandoglobulin® NF Liquid or Privigen®, treated every 3- or 4- weeks. Pooled analyses of raw patient data compared the rate of infection and other clinical outcomes during the course of the dosing cycle. Subjective symptoms of wear-off were quantified by comparing patient-reported overall well-being scores.

**Results:**

The probability of a first infection in the final week of the IVIG cycle was 1.26 (95 % confidence intervals [CI]: 0.76–2.11; *p* = 0.3621) and 1.55 (95 % CI: 1.04–2.32; *p* = 0.0314) times higher than in the first week, for patients on a 3-week cycle and 4-week dosing cycles, respectively. Wear-off, as manifested by a decrease in overall well-being, was experienced in 10 % of all cycles and reported at least once by 61 % of the patients on a 3-week cycle, and 43 % of those on a 4-week cycle.

**Conclusions:**

These findings confirm the existence of decreased efficacy (treatment wear-off) towards the end of a 3–4 week IVIG dosing cycle, and provide a quantifiable evaluation to a phenomenon typically reported anecdotally. For patients experiencing wear-off, increasing the IgG dose or shortening the dosing interval and/or a switch to SCIG may be beneficial.

## Introduction

Patients with antibody deficiency, a subclass of primary immunodeficiencies (PID), are predisposed to recurrent and persistent infections, requiring life-long, regular infusions of IgG to provide protection [1–3]. Intravenous IgG (IVIG) administered every 3- or 4- weeks has been the standard of care for patients with PID since the 1980s [4].

**Fig. 1:**
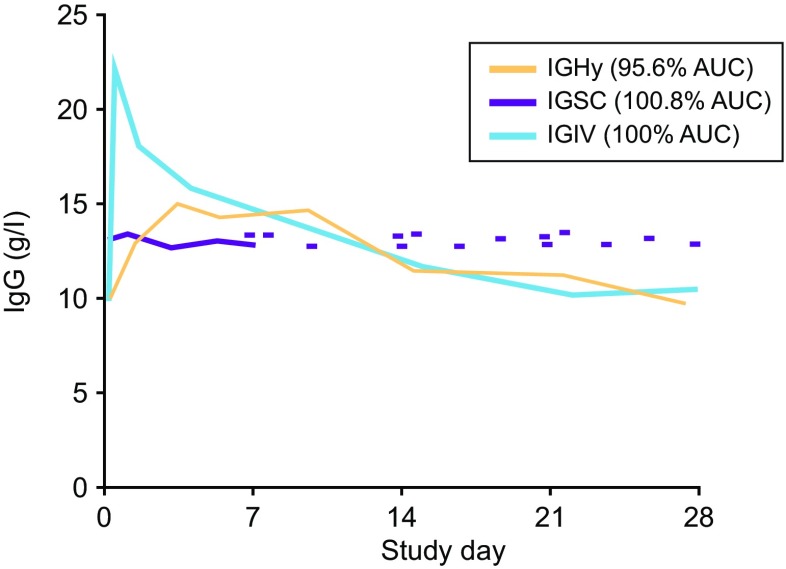
Serum IgG concentration following treatment by IVIG, SCIG and fSCIG. Representative pharmacokinetic serum concentration over time for a patient, comparing a 4-week intravenous infusion of IG (IGIV) with a weekly subcutaneous infusion at 143 % of the intravenous dose (IGSC) and a 4-week hyaluronidase facilitated SCIG dose at 104 % of the intravenous dose (IGHy). Area under the concentration curve (AUC) values are normalized to IGIV. Figure reproduced from fig. 5 in Wasserman et al. 2014 (adapted from fig. 1 in Wasserman et al. 2012), with kind permission of Elesevier Inc. [11, 12]

Infusion intervals of up to a month appear feasible due to IgG having a plasma half-life of approximately 26–41 days [5–8]. The IVIG infusion leads to a high peak in serum IgG concentration right after the end of infusion, followed by a rapid fall in the subsequent 48 h, and a slower decline over the next 30 days [9]. A more recently established alternative to IVIG is subcutaneous IgG (SCIG) [10]. SCIG is typically administered at more frequent intervals (daily to biweekly [every 2 weeks]), but infrequent dosing (up to every 4 weeks) is feasible with hyaluronidase facilitated SCIG (fSCIG) [11, 12]. Compared with IVIG, fSCIG does not produce a high post-infusion peak, but from the second week onwards decline in plasma IgG levels is similar (Fig. 1) [11, 12]. By contrast, frequent SCIG administration provides stable, ‘steady-state’, plasma IgG levels throughout the dosing cycle.

The “wear-off” effect in immune replacement therapy refers to the experience of diminished treatment efficacy towards the end of the 3–4 week IVIG treatment cycle, when serum IgG concentration may fall below a protective level. During this period, patients report an increased susceptibility to infection and a decrease in quality of life, clinically manifesting as general malaise, fatigue, arthralgia, and myalgias [9]. Wear-off is a common experience amongst patients. In the only survey we are aware to report the subjective feelings of wear-off (conducted in 2003), 42 % of the 1186 patients with PID reported feeling the effects of the infusion wearing off by the end of the treatment cycle as a typical experience of their therapy, while a further 26 % reported feeling wear-off occasionally [13].

Although acknowledged, accounts of wear-off effect are largely anecdotal, with few studies on this subject having been conducted to date. The aim of this investigation was to quantify the objective and subjective signs and symptoms of wear-off effect towards the end of the treatment cycle.

## Methods

### Patient Population

Results of three Phase III clinical trials of Sandoglobulin® NF Liquid (product no longer available, CSL Behring AG, Berne, Switzerland; NCT00168012) or Privigen® (CSL Behring AG, Berne, Switzerland; NCT00168025, NCT00322556) delivered every 3- or 4-weeks by intravenous administration were included in these pooled analyses. These multicenter trials were conducted in the United States and Canada (from September 2004 to January 2007). In all trials, the dosing interval was the same as the patients’ previous IVIG therapy, which had been selected as per standard of care, based upon their clinical response. Data from 130 patients with 7482 data points were used for the infection analysis, and data from 119 patients with 23,560 data points for the analysis of overall well-being. Demographic characteristics of patients enrolled in these studies are shown in Table 1.

**Table 1:** Demographic characteristics of patients from the pooled analysis^a^

Clinical study (ID)	Sandoglobulin® NF liquid (NCT00168012)	Privigen® (NCT00168025)	Privigen® (NCT00322556)
Total number of patients	42	80	55
Indication, *n* (%)			
CVID	32 (76.2)	59 (73.8)	44 (80.0)
XLA	10 (23.8)	21 (26.3)	11 (20.0)
Gender, *n* (%)			
Female	13 (31.0)	34 (42.5)	29 (52.7)
Male	29 (69.0)	46 (57.5)	26 (47.3)
Age (years), mean (SD)	32.2 (18.3)	28.2 (19.3)	29.9 (20.8)
BMI (kg/m^2^), mean (SD)	22.5 (5.1) (*n* = 38)	23.5 (6.3) (*n* = 80)	24.5 (7.8) (*n* = 53)
Duration of PID^b^ (years), mean (SD)	10.9 (8.8)	8.6 (7.5)	8.12 (6.8)
IgG trough level (g/L), mean (SD)	9.95 (2.69) (*n* = 41)	9.40 (2.75) (*n* = 79)	9.72 (2.23) (*n* = 54)
Median weekly dose (mg/kg)	125.5	117.8	128.2

*BMI* body mass index, *CVID* common variable immunodeficiency, *PID* primary immunodeficiency, *SD* standard deviation, *XLA* X-linked agammaglobulinemia

^a^Data for well-being are available only for a subset of patients

^b^Prior to study entry

All patients participating in the studies analysed here provided written informed consent. Approval from institutional review boards was obtained prior to the start of the studies.

### Study Endpoints

All endpoints and methods of their evaluation in the original studies were prespecified in the respective study protocols. The results reported here are from a *post hoc* retrospective pooled analysis of raw patient data.

The following clinical treatment efficacy endpoints were evaluated per week of the dosing cycle: 1) first occurrence of infection; 2) number of days with infection; 3) number of days hospitalized; 4) number of days off work/school; 5) number of days with fatigue.

Infections were identified in the study records as adverse events (AEs) with the system organ class “infections and infestations”, according to the Medical Dictionary for Regulatory Activities (MedDRA), current Version 18.0. Fatigue was identified by a search in the AE listings as any AE including the term “fatigue”. The number of days out of work/school was measured as the number of days out of work/school/kindergarten/day care or unable to perform normal activities due to the underlying PID or infection. The number of days hospitalized was assessed as the number of days hospitalized due to the underlying PID. Events for days out of work/school and days of hospitalization were recorded in patient diaries, which patients completed during their study participation. All patient data collected from Day 1 of the study until 48–96 h after the last infusion of the study, were used in the analyses. Patients were advised that a missing entry in the diary would be interpreted as no event. If the diary was not provided, the data were to be considered missing, but such case was not recorded for any of the diary data endpoints.

Subjective symptoms of wear-off were quantified by measuring the overall well-being of 119 patients enrolled in the studies NCT00168012 and NCT00168025. Of these patients, 33 were on a 3-week cycle and 86 on a 4-week cycle, representing a total number of 315 and 615 dosing cycles, respectively. Patients recorded daily their perception of overall well-being on a scale of 1–5, in which a score of 1 equated to very poor; 2, poor; 3, fair; 4, well; and a score of 5, very well. The clinical studies analyzed in this study were completed before the FDA guidance on patient-reported outcomes development (2009) [14] was published. To determine what could be considered a meaningful change in well-being score, a data variance analysis was performed. A drop of ≥1 point was considered clinically relevant, as it is approximately twice larger than between- or within-patient variance (0.403 and 0.437 for 3-week and 4-week regimens and 0.745 (3 week regimen) and 0.435 (4-week regimen), respectively).

### Statistical Analysis

Objective wear-off endpoints were analyzed by treatment cycle week using a generalized linear model for repeated count data within unique patients and compound symmetry correlation structure without any covariates. The actual time between infusions was accounted for in the model. Distribution analysis was performed using quasi-likelihood under the independence model criterion (QIC) [15].

Best fitting models were used to estimate the probability of a first infection and the number of days with fatigue, infection, hospitalization, and absence from work/school per week within the treatment cycle. The corresponding risk ratios vs. Week 1 were calculated. Analyses for the probabilities of infection, days off and hospitalization were additionally performed with time intervals shifted by 3 days (Week 1 covers Days 3–9; Week 2 covers Days 10–16; Week 3 covers Days 17–23; and Week 4 covers Days 24–31) based on the hypothesis that the average incubation period of the most common respiratory infections is approximately 3 days [16]. Binomial distribution was found to fit best for probability of first occurrence of infection, negative binomial distribution gave the best fit for the probability of number of days with infection and number of days with fatigue, and the Poisson distribution gave the best fit for probability of number of days hospitalized and number of days off work/school, per week of the treatment cycle. The impact of median IgG trough levels during study, categorical patient age (2–11, 12–15, 16–64, and ≥65 years), PID diagnosis (X-linked agammaglobulinemia [XLA] versus common variable immunodeficiency [CVID]), and presence or absence of bronchiectasis at study entrance, was evaluated in relation to the study endpoints using a generalized linear model for repeated measures.

Overall well-being scores were analyzed using descriptive statistics. Patient reported wear-off (as manifested by decreased well-being) was defined for every patient as a drop in overall well-being of ≥1 on >3 days of the last week of a dosing cycle compared with the mean score recorded in Week 2. No imputation was performed for missing data for overall well-being. A total of 8 out of 23,568 data points (0.034 %) in 3 patients were missing. The influence of these missing data points was considered extremely low and thus excluded from analysis.

No adjustment of *p*-values for multiplicity was done in this *post hoc* analysis.

## Results

### Patient Population

Data were collated from three Phase III clinical trials of IVIG therapy in patients with PID providing a total sample size larger than the majority of single studies in patients with PID.

The majority of patients in each of the three studies was diagnosed as having CVID, and on average the duration of PID before study entry was 8–11 years. Patients on a 3-week cycle had only slightly higher average serum IgG trough levels, although they received notably higher average weekly IgG doses than those on a 4-week cycle (Table 2). The median number of dosing cycles recorded in the study was 7 (range: 1–38) for the 3-week cycle, and 6 (range: 1–31) for the 4-week cycle. The median number of dosing cycles for the set of 119 patients for which overall well-being scores were assessed, was 8 (range: 3–15) for the 3-week cycle, and 8 (range: 1–12) for the 4-week cycle.

**Table 2:** Comparison of IgG trough levels for 3- and 4-week dosing cycles

	3-week cycle			4-week cycle		
	Mean (SD)	Minimum	Maximum	Mean (SD)	Minimum	Maximum
Median weekly dose (mg/kg)	168.1 (48.61)	95.2	266.7	118.70 (33.91)	50	222.0
Median IgG trough level (g/L)	10.8 (2.91)	5.5	18.0	9.2 (2.3)	4.9	21.9

*SD*, standard deviation

### Occurrence of Infection

**Fig. 2:**
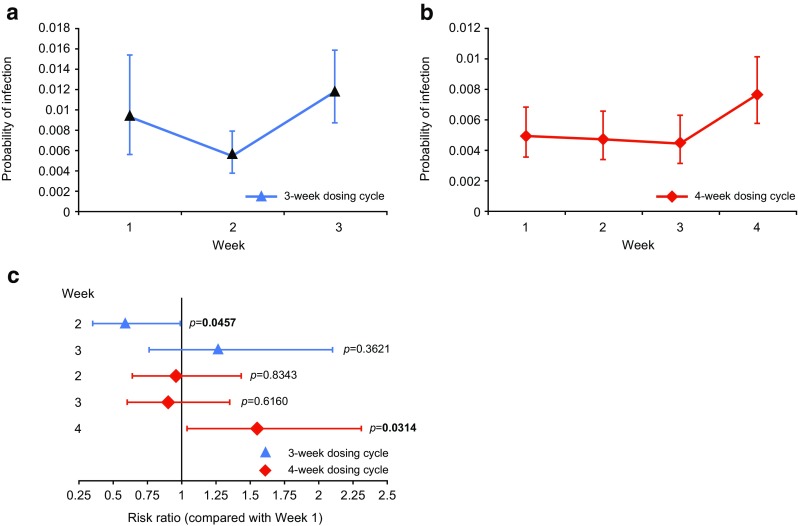
Probability of first infection per week within the dosing cycle. The probability of a first infection at each week of a 3-week (**a**), or 4-week (**b**) dosing cycle was determined by binomial distribution: (**c**) Risk ratio of a first infection during Week 1 of the dosing cycle compared with subsequent weeks. Error bars indicate 95 % confidence intervals (CI)

The most common infections experienced by patients were sinusitis, nasopharyngitis and upper respiratory tract infections. The probability of a first infection during a dosing cycle was significantly increased in the last week of the 4-week dosing cycle. There was also an increased probability of a first infection in the last week of the 3-week dosing cycle, although this did not reach statistical significance (Fig. 2). Compared with Week 1, the risk ratio for a first infection during the final cycle week was 1.26 (*p* = 0.3621; 95 % confidence interval [CI] 0.762, 2.105) and 1.55 (*p* = 0.0314; CI 1.04, 2.316) for patients on 3- and 4-week dosing schedules, respectively. A generalized linear model for repeated measures showed that higher IgG trough levels were associated with a slightly increased risk for infections throughout the cycle (*p* = 0.034 for 3-week and *p* = 0.26 for 4-week dosing cycles). Among patients on a 4-week dosing cycle, those with a diagnosis of CVID were also at increased risk for infections (*p* = 0.003). However, these factors had only a minimal impact on the risk ratio for infection in the final week compared with Week 1.

**Fig. 3:**
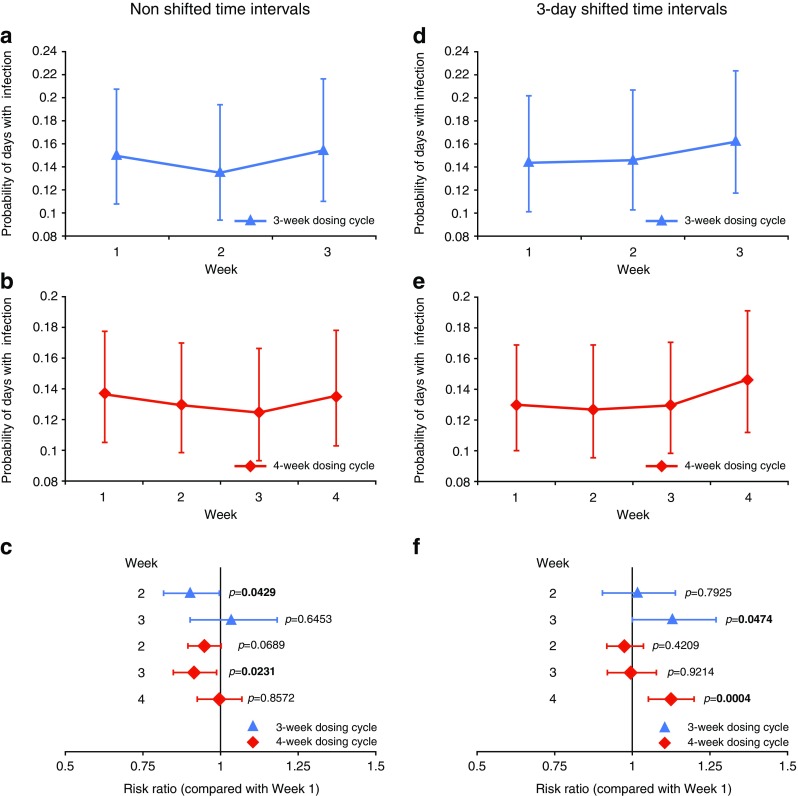
Probability of days with infection per week within the dosing cycle. The probability of days with infection, as determined by negative binomial distribution, is shown for each week of the 3-week (**a** and **d**) and 4-week (**b** and **e**) dosing cycles using non shifted (**a** and **b**) or shifted (**d** and **e**) time intervals; risk ratio of a day with infection during Week 1 of the dosing cycle compared with subsequent weeks using non shifted (**c**) and shifted (**f**) time intervals. Time intervals were shifted by 3 days to account for infection incubation period: Week 1 covers Days 3–9; Week 2 covers Days 10–16; Week 3 covers Days 17–23; and Week 4 covers Days 24–31. Error bars indicate 95 % CI

The probability of days with infection was significantly lower in the second and third weeks of the 3- and 4- week dosing cycles, respectively (Fig. 3a–c). Shifted time interval analysis (accounting for the average incubation period of infection) showed that the probability of infection was greatest during the last week of the cycle for both 3- and 4- week cycles, with a significantly greater risk compared with Week 1 (1.13, *p* = 0.0474, and 1.13, *p* = 0.0004, times, respectively) (Fig. 3d–f).

### Fatigue

**Fig. 4:**
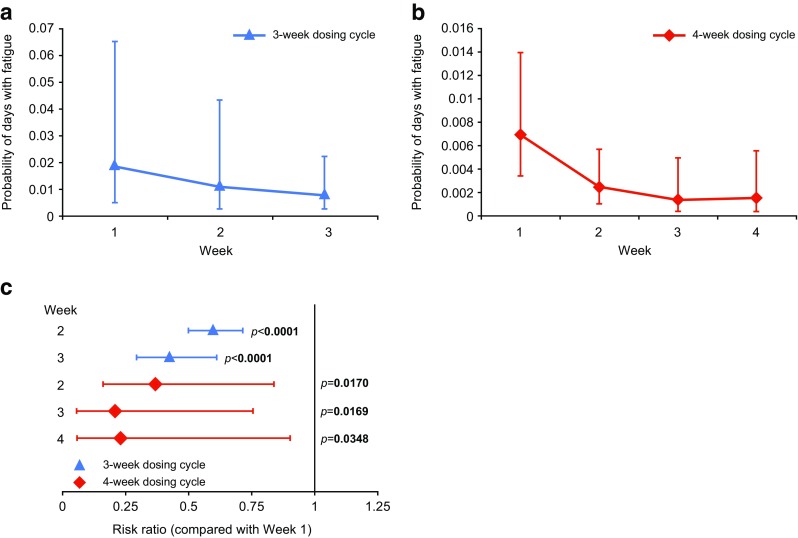
Probability of fatigue. Probability of days with fatigue, as determined by Poisson distribution at each week of a 3-week (**a**), or 4-week (**b**) dosing cycle is shown; risk ratio of fatigue during Week 1 of the dosing cycle compared with subsequent weeks (**c**). Error bars indicate 95 % CI

The probability of fatigue was greatest in the first week of both the 3- and 4-week dosing cycles, and was significantly lower in the subsequent weeks of the treatment cycle (Fig. 4). Compared with Week 1, the risk ratio for fatigue in subsequent weeks was at most 0.595 (*p* < 0.0001) and 0.367 (*p* = 0.0170) for patients on 3- and 4-week dosing schedules, respectively.

### Hospitalization or Absence from Work/School

The average number of days with hospitalization or days off work/school were not significantly different across the cycle weeks for either dosing cycle. The number of events (days/patient/week) for hospitalization for each week of the cycle ranged from 0.005 to 0.024 and from 0.028 to 0.048 for 3- and 4- week dosing cycles, respectively. The corresponding values for days off work/school were 0.274–0.358 and 0.114–0.191, respectively. A generalized linear model for repeated measures suggests that patients diagnosed with CVID have more days with hospitalization and/or days off work/school than those patients diagnosed with XLA. Additionally, patients with bronchiectasis on a 4-week dosing cycle had a higher probability of experiencing days off work/school etc. (unable to perform normal daily activities), compared with patients without comorbidity of bronchiectasis (*p* = 0.0159 for unshifted analysis and *p* = 0.0169 for analysis shifted by 3 days).

### Patients’ Overall Well-Being

**Fig. 5:**
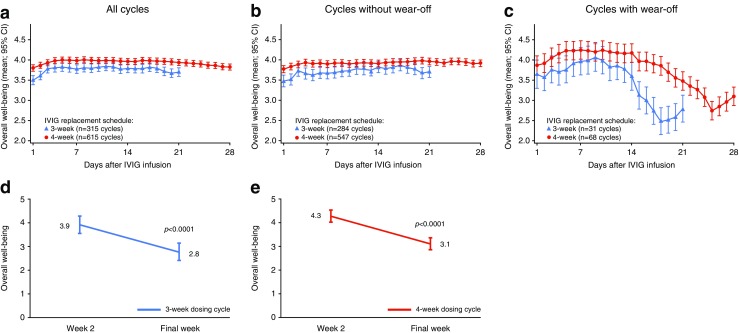
Patient-reported overall well-being during IVIG dosing cycle. Mean average (95 % CI) reported by patients each day of a 3-week cycle (*blue line*) or a 4-week cycle (*red line*) for all treatment cycles (**a**), those cycles in which no wear-off was reported (**b**), and those in which wear-off was reported (**c**). Change in overall well-being in Week 2 and the final week of a 3-week (**d**), or a 4-week (**e**) cycle during treatment cycles in which wear-off was reported. Score represents: very poor (1), poor (2), fair (3), well (4), and very well (5). Error bars indicate 95 % CI

Scores for overall well-being amongst the study patients indicated a general feeling of being ‘well’ for patients on both 3-week and 4-week IVIG dosing cycles (Fig. 5a). Patients on a 4-week dosing cycle had, on average, higher overall well-being, but the course of change was similar between the dosing cycles, with the lowest values registered during the last pre-infusion week of the dosing schedule. After dosing, there were daily score increases until Day 5, from 3.5 to 3.8 (3-week cycle) and from 3.8 to 4 (4-week cycle). Scores then remained stable until the end of Week 2, whereupon there was a steady drop until the end of the cycle to 3.7 and 3.8 for patients on 3-week and 4-week cycles, respectively (unadjusted *p*-value from paired t-test 0.0001 for both schedules).

Twenty patients on a 3-week cycle (61 %) and 37 patients on a 4-week cycle (43 %) reported symptoms of wear-off at least once, as defined by a drop in overall well-being score of ≥1 point on >3 days of a dosing cycle’s last week compared with the mean score recorded in Week 2. Wear-off, as manifested by well-being decrease, was reported in 10 % of all cycles. Summarizing these cycles, patients on a 3-week cycle experienced a steady increase in overall well-being from 3.6 to 4.1 by Day 9, and then a sharp decline to 2.5 by Day 18 (Fig. 5b). For patients on a 4-week cycle, there was steady score increase from 3.8 after dosing to 4.2 by Day 5. Scores remained stable until the end of Week 2 and then decreased rapidly to 2.8 in the final week of the cycle. Mean overall well-being decreases of 1.2 were recorded from Week 2 to the final week of the dosing cycle for both the 3- and 4-week cycles (*p* < 0.0001) (Fig. 5d and e).

Among patients who did not experience wear-off, there was a slight increase in overall well-being in the 3–4 first days of the cycle (Fig. 5b), thereafter, no characteristic change was seen.

## Discussion

The aim of IgG therapy is to protect patients with PID against infections by providing adequate serum and tissue concentrations of IgG. However, a universal protective IgG concentration does not exist and varies from individual to individual depending on a number of internal and external factors. The term “biologic IgG level” was coined to reflect an individual’s protective IgG concentration [17] and it is the aim of the treatment to identify and maintain IgG concentrations above this level. In healthy adults, 7 g/L represents a value within the normal plasma IgG range (6–16 g/L) and is, therefore, often considered to be associated with reasonable protection against infection in many immunocompetent individuals and patients with PID [18–20]. As IgG concentrations decline during the IVIG dosing cycle, patients report a feeling of treatment wear-off towards the end of their cycle, which may reflect IgG concentrations dropping below biologic levels.

In quantifying objective wear-off outcomes, we found that the first occurrence of infection and number of days with infection per week of IVIG infusion cycle, provided a useful objective measure for the efficacy of therapy at each stage (week) of the treatment cycle. During the final week of the treatment cycle, when serum IgG levels approached their trough, the probability of infection increased for PID patients on both 3- and 4-week dosing cycles.

We also noted that infections appeared to be more frequent during the 3 days following the infusion than the rest of Week 1 and Week 2. This led to the hypothesis that these infections might have started developing just before the next IVIG infusion, since common infections have an incubation period of a few days. We tested this hypothesis by performing a ‘shifted’ analysis (i.e. the days of infection were shifted by 3 days). These analyses showed a clearer accumulation of infections during the final week of the dosing cycle, supporting the notion that when analyzing wear-off effect, reasonable incubation periods of infection should be taken into consideration.

A generalized linear model for repeated measures, including median IgG trough levels as a covariate, found that patients with higher trough levels tended to have slightly more infections. A possible explanation is that patients with more severe PID condition received higher doses of IgG therapy, leading to higher trough levels. However, their individual protective serum IgG levels (“biologic levels” according to Bonagura et al. [17] were apparently higher as well).

Objective signs of wear-off in the last pre-infusion week were not observed for number of days off work/school, or number of days of hospitalization. These events were rare and therefore less informative for the purposes of this analysis.

In the absence of a standard measure of patient reported wear-off, using an overall well-being endpoint, we introduced a conservative threshold of well-being declining by at least 1 point on the 5-grade scale. The observed within-patient and between-patient data variances suggest this value to be a conservative threshold. With respect to patients’ subjective perception of treatment wear-off defined this way, results from daily records have indicated that 42 % of patients experienced the feeling of wear-off in the second half of the dosing cycle on a regular basis [13]. We found that patient-reported overall well-being proved to be an important quantitative indicator of treatment wear-off effect. A measurable decline in well-being was experienced by patients during the final week of the dosing cycle. On average wear-off was experienced in 10 % of all cycles, suggesting that wear-off, or its perception, is influenced by factors beyond serum IgG levels, including random events such as exposure to infectious agents. For those on a 4-week cycle, this decline appears to originate from the second half of the third week. In patients experiencing wear-off, some improvement was observed a few days before the next infusion, which is difficult to explain, unless it is a psychological effect of the improvement expected with the upcoming infusion.

In addition to measuring wear-off, patients’ perception of well-being also reflects the impact of receiving an IVIG infusion: reported well-being was lowest just after the administration of IVIG, improving over the next 2–3 days. This most likely reflects the impact of known systemic AEs of IVIG therapy, associated with peak serum protein concentration immediately after IVIG infusion (fever, headache, and nausea) [9, 21]. Alternatively, the low well-being in the first week of the cycle may be a delayed result of an infection experienced during the previous week, but this would be difficult to evaluate. Of note, study patients included in this analysis had a broad range of median IgG trough levels (4.9–21.9 g/L; Table 2), extending beyond the lower and upper thresholds of the normal plasma IgG range (6–16 g/L [18–20]); this observation reflects the efforts to achieve individual protective IgG concentrations and thus minimize the risk of infections.

Fatigue is another adverse event commonly associated with IVIG infusion [21]. It is therefore unsurprising that fatigue was most likely to be experienced during the first week post IVIG infusion in an unshifted anlaysis. Fatigue has been described as a sign of wear-off [9]. However, the present investigation does not support this hypothesis, as an increase in the incidence of fatigue towards the end of the cycle was not observed.

Overall, results from our analyses illustrate that patients with PID on IVIG therapy regularly experienced decreased feeling of overall well-being on both ends of the dosing cycle: for the first several days right after the IVIG infusion (likely due to a combination of side effects of IVIG and infections carrying over from the previous cycle), and during the last week of the dosing cycle (wear-off).

Wear-off has also been reported during the use of IVIG to treat neurologic diseases. Neurologists have anecdotally reported patients experiencing diminished strength and ability to perform daily tasks towards the end of their dosing cycles [22]. In a case report of a patient with multifocal motor neuropathy, sharp fluctuations in strength (Medical Research Council score for muscle strength) were observed during the 4-week cycle, with strength peaking after each infusion [23]. Subsequently, strength was stabilized after the patient was switched to weekly SCIG infusions.

A limitation of this study is that it represents a retrospective pooled analysis of data from several trials. Prospective trials would be warranted to confirm the results presented here. On the other hand, pooling retrospective data into a combined dataset allowed using a higher number of patients and data points than a single PID study can provide, and inspires confidence that the analyses reported here can be considered conclusive.

Allocation of patients to a 3- or 4-week dosing cycle was based on physicians’ clinical judgment and reflects standard clinical practice. Patients on a 3-week cycle received approximately 40 % higher doses than those on a 4-week cycle (weekly equivalent of the mean of median doses of 168.1 mg/kg vs. 118.7 mg/kg, respectively), but the IgG trough levels were only slightly higher (mean of median troughs at 10.8 g/L and 9.2 g/L, respectively), suggesting that patients on a 3-week cycle were metabolizing IgG faster. In these patients, the overall numbers of first infections, days with infection, and the probability of infection were also higher, suggesting that they were more vulnerable to infection. Therefore, it appears that the higher IVIG doses and resulting higher serum IgG levels experienced by these patients did not ensure the same protection against infections compared with the 4-week dosing group. Consistent with these observations, patients on a 3-week cycle reported a lower degree of well-being throughout the cycle and experienced a sharper decrease in overall well-being at the end of the dosing cycle than those receiving IVIG on a 4-week cycle. A higher proportion of these patients experienced patient-reported wear-off, when compared with patients on a 4-week cycle. It is likely that patients were assigned to a 3-week dosing cycle owing to greater PID severity. However, this strategy appears to have been only partially successful. A solution for avoiding wear-off would be to decrease the IVIG dosing interval to less than 3 weeks. However, this may not be convenient for patients receiving IVIG administration at a hospital or clinic, and would incur increased healthcare costs as a consequence. More frequent SCIG infusions – daily, several times per week, weekly, or biweekly – offer a convenient alternative, which can be administered at home (it is worth noting that IVIG can also be administered at home, but this is not a popular option in many countries, and it usually requires visit of a home infusion nurse). Dividing the single monthly IgG dose into smaller, but more frequently administered doses, results in more stable serum IgG levels with reduced peak/trough variation [5, 9, 24]. As a result, patients do not experience diminished serum IgG levels towards the end of their dosing cycle, and consequently should not be subject to treatment wear-off. Although there is no direct evidence of the wear-off effect for the recently introduced treatment modality of fSCIG (characterized by typical IVIG infusion cycles every 3–4 weeks [11]) it would be important to study if the IVIG-like pharmacokinetic features of this therapy are also associated with symptoms or signs of wear-off.

Through this study we have been able to quantify the wear-off effect in terms of both clinical response (increased frequency of a first infection during the last week of the IVIG cycle, and increased number of infection days during this period) and patient perception (successive drop in overall well-being during the final week of the IVIG cycle). Both clinical and patient-reported outcomes of wear-off appear to be associated with the dropping IgG concentration at the end of the dosing cycle.

## Conclusions

Pooled data analyses allowed quantifying the objective and subjective symptoms and signs of IVIG treatment wear-off effect. For patients experiencing wear-off, increasing the IgG dose, shortening the dosing interval, and/or a switch to SCIG may be beneficial.
